# A retrospective analysis of eleven cases of invasive rhino-orbito-cerebral mucormycosis presented with orbital apex syndrome initially

**DOI:** 10.1186/s12886-016-0189-1

**Published:** 2016-01-12

**Authors:** Nan Jiang, Guiqiu Zhao, Shanshan Yang, Jing Lin, Liting Hu, Chengye Che, Qian Wang, Qiang Xu

**Affiliations:** Department of Ophthalmology, the Affiliated Hospital of Qingdao University, Qingdao, Shandong Province China

**Keywords:** Rhino-orbito-cerebral mucormycosis, Orbital apex syndrome, Ocular manifestations, Diabetes mellitus, Immunocompromise

## Abstract

**Background:**

Rhino-orbito-cerebral mucormycosis(ROCM) is an invasive fungal infection that usually occurs in immunocompromised patients and sometimes presents as orbital apex syndrome(OAS) initially. It is rapidly fatal without an early diagnosis and treatment. We report the cases of invasive ROCM presenting with OAS initially in order to raise the attention of clinicians.

**Methods:**

We retrospectively investigated eleven cases of invasive ROCM presenting initially with OAS admitted between January 2006 and December 2013. We analyzed clinical features, results of laboratory and radiological examinations, nasal endoscopy, aggressive surgical excision and debridement, and medical management outcomes of each case.

**Results:**

A total of eleven cases of invasive ROCM with OAS as an initial sign were presented. Mucormycosis was accompanied by type II diabetes mellitus in nine cases, renal transplant in one case, and injury caused by traffic accident in one case. Anterior rhinoscopy revealed palatine or nasal necrotic lesions in all patients, and transethmoidal optic nerve decompression was carried out in three patients at the same time. CT scan revealed rhino-orbital-cerebral involvement in every patient. All patients were given intravenous amphotericin B. Nine patients underwent surgical debridement of necrotic tissue. Three patients survived.

**Conclusions:**

ROCM is a severe, emergent and fatal infection requiring multidisciplinary management. It may often present with OAS initially. For ophthalmologist, mucormycosis must be considered in immunocompromised patients presenting with OAS initially, and anterior rhinoscopy is imperative before hormonotherapy, even in the cases absent of ketoacidosis induced by diabetes mellitus.

## Background

Mucormycosis is a rare and severe opportunistic fungal infection resulted from a fungus of the order mucorales. At present, the incidence of fungal infections is increasing. Mucormycosis is the second most frequent fungal infection following aspergillus [1–3]. This fungal infection can rapidly progress in individuals who are immunologically or metabolically compromised through vascular thrombosis or central nervous system involvement [4]. The most common co-morbidities include diabetes mellitus, lymphoid malignancy, burn, severe trauma, renal failure and steroid theraphy [5, 6].

The rhino-orbital-cerebral presentation is the most frequent. The diagnosis is often made late and relies on anatomopathological and mycological examinations, especially for some patients presenting with atypical symptoms initially for example swelling lid, nasal stuffiness, vision loss *et al*. Rhino-orbital-cerebral mucormycosis (ROCM) may be rapidly fatal if not recognized early and treated promptly. Early diagnosis and appropriate medical management are vital to save the life. In this paper, we retrospectively presented eleven cases of ROCM with ocular symptoms initially and discussed clinical features, results of laboratory investigations, radiological examinations and treatment outcomes of each case.

## Methods

Eleven ROCM patients with orbital apex syndrome(OAS) initially presenting to our hospital between January 2006 and December 2013 were retrospectively investigated. These studies were conducted in full conformance with the principles of the Declaration of Helsinki in 1995 or with the laws of China, which ever afforded the greater protection to the study participant. Written informed consents were obtained from the Institutional Research Ethics Committee at the Affiliated Hospital of Qingdao University and eleven patients for publication of this research article and any accompanying images.

Evaluation at presentation included a detailed history, clinical signs, ENT, ophthalmic, and neurological examination to assess the extent of disease, nasal endoscopy with biopsy, and results of laboratory and radiological examinations.

The diagnosis of mucormycosis was made by histological examination of biopsy samples and finding filaments of the Mucorales order. CT and MRI scan revealed rhino-orbital-cerebral involvement in every patient. Treatment with systemic amphotericin B was started as soon as the diagnosis of mucormycosis was established. Treatment was also instituted to stabilize the underlying metabolic derangement and surgical debridement was indicated.

## Results

Out of 11 cases of ROCM followed and treated in our hospital, three were female and eight were male. The mean age of the patients was 53.7 years (range: 45–60 years). Diabetes was the most frequent risk factor, presenting in nine patients. One patient presented with renal transplant, and one was injured by traffic accident .Of 11 cases of mucormycosis with rhino-orbito-cerebral involvement, all had OAS. Vision was altered in all patients including blindness in eight and only perception of light/hand moving in three cases.

**Fig. 1:**
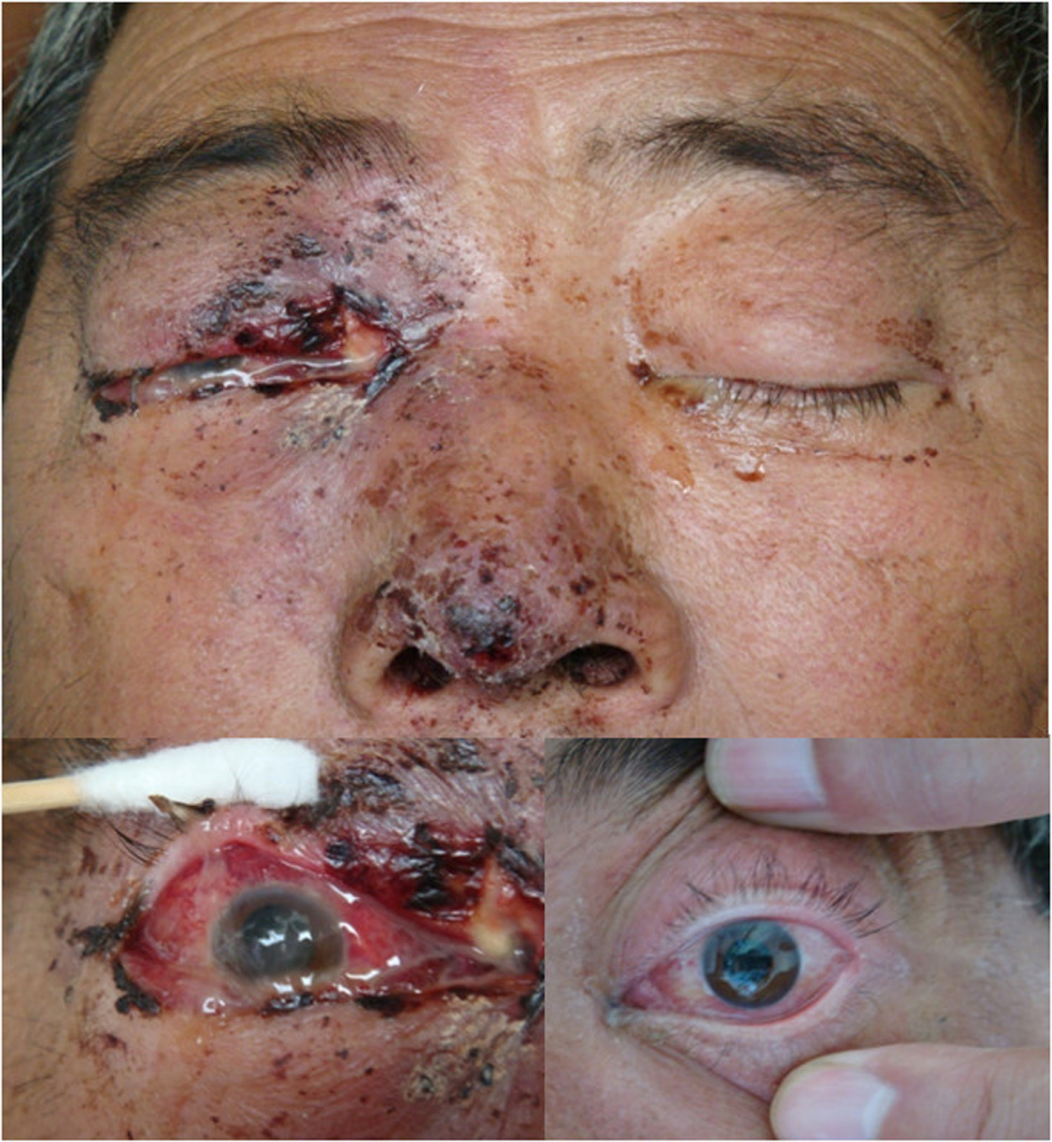
Pre-operative aspect of patient exhibiting bilateral periorbital edema with facial swelling, exophthalmia, blepharoptosis,and Ocular purulent secretion

The main clinical signs on physical examination were progressively decreased vision (*n =* 11), involvement of cranial nerves (*n =* 11), blepharoptosis (*n =* 11), exophthalmia (*n =* 9), periorbital edema (*n =* 9), and facial swelling (*n =* 8) (Fig. 1).

Table 1 shows main clinical features, locations of the involvements, imaging manifestation, and therapeutic data of the cases.

**Table 1:** main clinical features, locations of the involvements, imaging manifestation, and therapeutic data of the cases

	N
Cases	11
Mean age (range)	53.7 (45–60)
Gender(F/M)	3/8
Accompanying disease	
Diabetes mellitus	9
Renal transplant	1
Trauma	1
Locations of the involvements	
Rhinocerebral	11
Sino-orbital	0
Rhino-orbital-cerebral	11
Clinical involvement	
Orbital apex syndrome	11
Imaging manifestation(CT/MRI)	
Thickening in sinus Mucosa	11
Inflammation in the periorbital muscles	11
Involvement of the cavernous sinus	4
Occlusion of the internal carotid artery	2
Sings of cerebral infarct	2
Rhinoscopy	11
Therapy	
Surgical debridement	8
Amphotericin B.	11
Outcomes	
Survive with sequeale	3
Death	8

Table 2 shows clinical signs and symptoms of the cases of mucormycosis.

**Table 2:** Clinical features and radiological features of the cases of mucormycosis

Signs/symptoms	n	%
Fever	9	81.8
Headache	7	63.6
Consciousness	6	54.5
Cranial nerve palsy	11	100
Decreased vision	11	100
Exophthalmia	9	81.8
Diplopia	9	81.8
Blepharoptosis	11	100
Periorbital edema	9	81.8
Facial swelling and pain	8	72.7
Ocular purulent secretion	7	63.6
Nasal blockage/crusting	6	54.5
Blood stained discharge	5	45.5

**Fig. 2:**
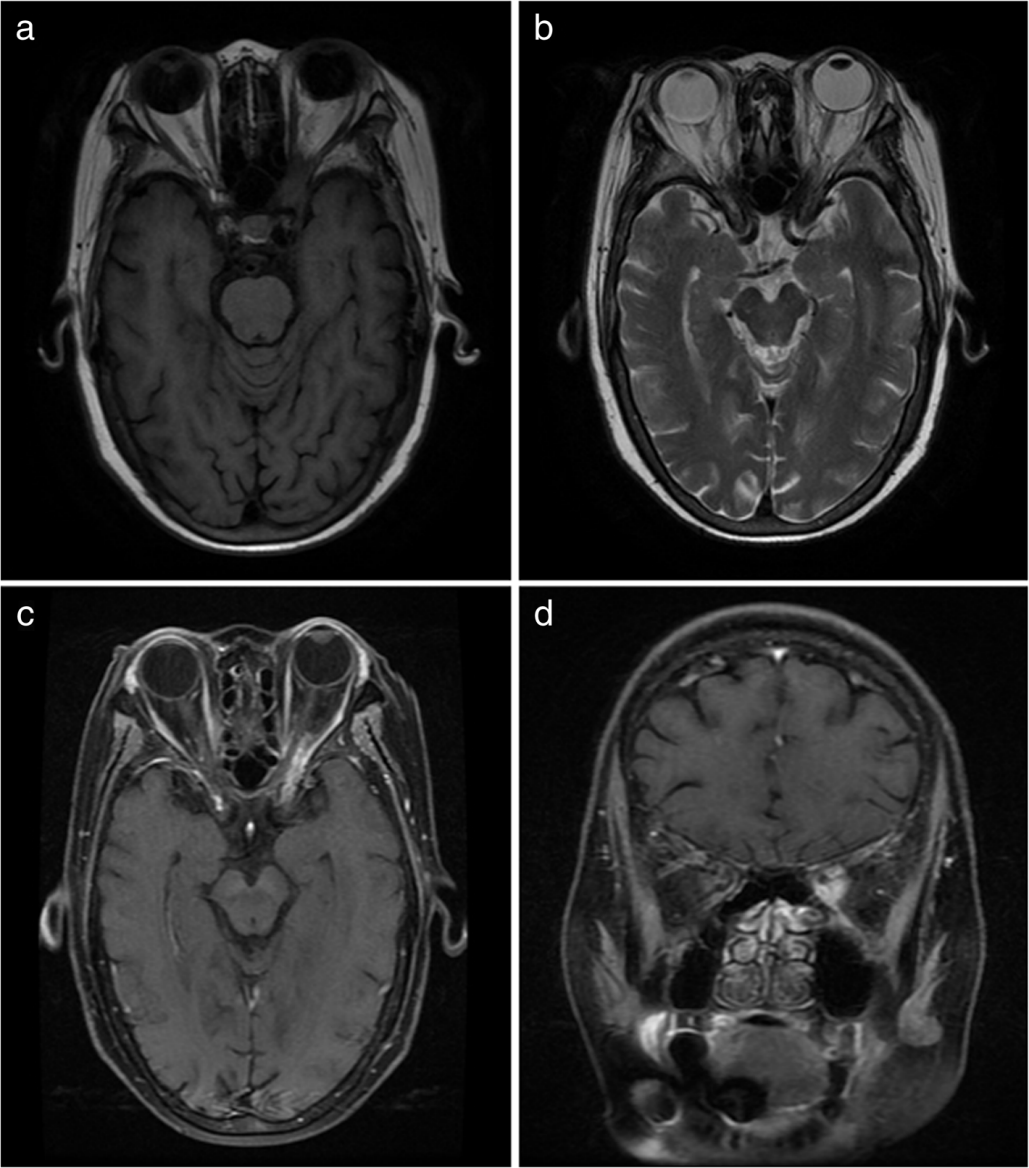
MRI presentations of mucormycosis presented with orbital apex syndrome. **a**: Axial T1WI MR shows a isointensity lesion in the left orbital apex;**b**: Axial T2WI MR shows a hypointensity lesion in the left orbital apex and high signal in the sphenoid sinus; **c**: Axial contrast-enhanced T1WI MR shows a enhancing lesion in the left orbital apex; **d**: Coronal contrast-enhanced T1WI MR shows a enhancing lesion in the left orbital apex

All the patients underwent CT/MRI scan of the paranasal sinuses, orbit and craniocerebrum. Pansinusitis was found in eight patients, and maxilloethmoidal sinusitis was found in three patients. The thickening sinus mucosa and extension of lesions to the orbits were observed in all patients. Four patients presented with thrombosis of cavernous sinuses. Extensionto frontal lobes, dilatation of the ventricular system and cortical grooves, and thrombophlebitis of the right lateral sinus were observed in one patient each. MRI performed in one patient revealed lesion predominantly located in sphenoid sinus, involving the left orbital apex (Fig. 2).

**Fig. 3:**
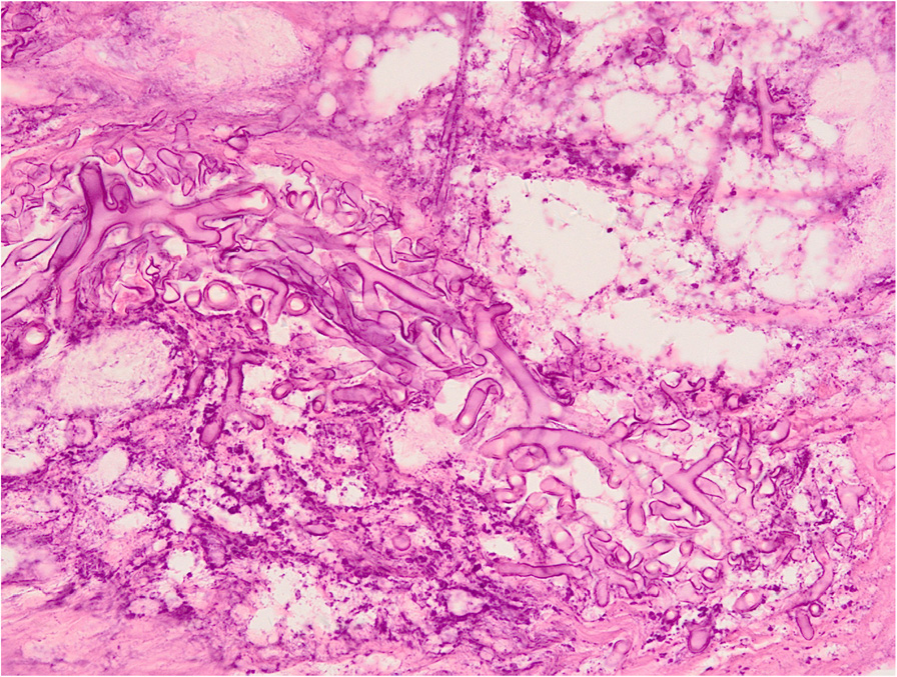
Nasal biopsy showing mycelial filaments of variable thickness and necrosis (PAS;×200)

On anterior rhinoscopy all patients had black blood stained debris in the region of inferior and middle turbinate along with necrosis on endoscopy. Direct examination was performed after PAS staining. Tissue specimens for culture were obtained from all cases. The culture was made on Sabouraud chloramphenicol medium with and without cycloheximide. In ten cases, the diagnosis of mucormycosis was based on positive histopathological findings for mucormycosis (Fig. 3). In three cases, Mucor spp. was isolated from tissue cultures and it was pathologically verified in all patients of mucormycosis. The histological examination revealed large non-septate mycelial filaments, sometimes branched at a right angle with images of angioinvasion within necrotic and inflammatory material.

Duration of illness between the first clinical signs and diagnosis ranged from 10 days to 50 days. At the early stage, the nasal and sinus phase was neglected by all patients. Once diagnosis, all patients had received antifungal treatment with amphotericin B, at 0.3 mg/kg per day initially with close renal function monitoring, gradually up to maximum dose at 1 mg/kg per day for an average of 4–6 weeks. During the course, three patients presented with severe renal insufficiency induced by amphotericin B treatment leading to treatment interruption and switched to itraconazole. For the patients accompanied with diabetes mellitus or renal transplantation, insulin therapy with a strict surveillance of glycemia or the therapy with improvement of renal function was prescribed. Meanwhile, nine cases received surgical debridement of necrotic tissue associated to drug therapy.

Finally, one patient with trauma had been rapidly transferred to the ICU for severe skin and soft tissue infection, and died of septicemia and acute respiratory insufficiency. The patient with renal transplantation refused further treatment and died of renal failure. The outcome was favorable for three patients with diabetes mellitus, 12, 18, and 24 months later, respectively. Three patients had fulminating disease and early mortality due to intracranial and pulmonary infection. One died of ventricular arrhythmia disorder, one died of neurological distress, and one case was due to hemorrhagic shock.

## Discussion

Mucormycosis is a rare filamentous and fatal fungal infection mostly encountered among immuno-suppressed patients [7, 8]. The most common risk factors accompanied with mucormycosis are diabetes mellitus, especially ketoacidosis, immunosuppressive conditions like hematologic malignancies and organ transplantations [9–11]. Nine patients (81.8 %) had diabetes mellitus, in which neutrophils had an impaired ability of phagocytosis and chemotaxis [12]. Presence of diabetic ketoacidosis increases predisposition to mucormycosis. Acidosis disrupts iron binding of transferrin, resulting in increased proportion of unbound iron, which may promote growth of the fungus [13, 14]. Another accompanying disease is renal transplantation. The least frequently accompanying disease is haematological malignity.

Mucormycosis presents in various distinct forms depending on the immunological status of the host and the affected site. Among the various localizations, the rhino-orbital-cerebral presentation is the most frequent (40 %) [15, 16]. The fungus invades the wall of the blood vessels, causing mechanical and toxic damage to the intima leading to thrombosis, and later it invades the lymphatics and veins. These thromboses cause emboli and vascular obstruction responsible for tissue necrosis.

The infection progressively spreads from the nasal mucosa to the nose, facial sinuses, the palate, orbits and brain. It evolves in three stages: nasal and sinus involvement often pauci or asymptomatic unnoticed by the patient, orbital involvement motivating the patient for consultation, and finally cerebral involvement.

OAS is a rare syndrome with retro-orbital pain, complete ophthalmoplegia, and visual loss. In the etiology of OAS, inflammatory, infectious, neoplastic, iatrogenic/traumatic and vascular pathologies should be considered [17]. Fungal infections can cause extensive tissue damage potentially leading to permanent vision loss and death if not treated [18]. Species of Fusarium, Aspergillus, Candida, dematiaceous fungi, Mucorales and Scedosporium predominate. Diagnosis is aided by recognition of typical clinical features and by direct microscopic detection of fungi in scrapes, biopsy specimens, and other samples. Culture confirms the diagnosis. Histopathological, immunohistochemical, or DNA-based tests may also be needed [19]. For example, ROCM, also known as mucormycosis, is commonly caused by the non-septate filamentous fungus, Rhizopus oryzae [20]. Patients with ROCM present acutely and progress rapidly. Another fungal orbital infection important to understand is sino-orbital aspergillosis. Sino-orbital Aspergillus infection can occur acutely or chronically and can affect both the immunocompetent and immunocompromised [21, 22]. Invasive Aspergillus infection in the immunocompetent host usually presents in a more indolent but progressive course. CT imaging can show heterogenous soft tissue enhancement with focal bony destruction with intraluminal calcification being indicative of an Aspergillus infection [23, 24].

For mucormycosis with a late diagnosis, the evolution is marked by extension to the orbits. The orbital involvement is related to the vascular tropism of the fungus inducing arteriole thrombosis affecting the orbit wall, oculomotor and optical nerves responsible for blindness with or without thrombosis of the ophthalmic artery. It is unilateral and marked by orbital pain, diplopia, ophthalmoplegia, periorbital edema, chemosis, exophthalmia or even blindness. OAS may be firstly diagnosed. Eye fundus is a key examination for the diagnosis of ROCM. It may reveal a venous congestion or thrombosis of the artery or of the central vein of retina, optic atrophy or panophthalmia [16, 25–27]. In the OAS, the prognosis is good if treated early. There is mucosal invasion and hence radical debridement and antifungal therapy is imperative. If the inflammation can’t be controlled, intracranial involvement occurs from invasion by way of superior orbital fissure, ophthalmic vessels, cribriform plate and not uncommonly, through carotid artery.

In the patients involved in our study, the clinical signs and symptoms of mucormycosis may vary with the involved organ widely. Mostly, the clinical presentation is atypical. Nasal discharge or nasal blockage may occur in a small proportion of the patients. Sometimes even eschar, typical presentation of rinocerebral mucormycosis may not appear. The patients reported here most frequently presented with ptosis, exophthalmia and examination of the revealed sinuses eschar in most of cases. In such patients, MRI has the advantage of detecting early vascular and intracranial invasion.

Successful treatment of mucormycosis is based on three principles. First, early diagnosis is greatly imperative and can enormously reduce the patient’s mortality. Second, necrotic tissues should be aggressively debrided or infected tissues should be resected. Last, medical treatment with antimycotic agents should be carried out [7]. Surgical therapy alone has a great influence on treatment outcome in cases of mucormycosis. In fact, Tedder *et al.* reported that the mortality rate was 11 % in patients who underwent surgery but 60 % in patients without surgical therapy [28]. Systemic antifungal therapy includes the use of high dose amphotericin B and is associated with an overall survival rate of 72 %. It is usually given in dextrose 5 % in water intravenously at a dose of 1.0–1.5 mg/kg daily since its usage is associated with renal toxicity and it requires careful monitoring of serum urea nitrogen, blood urea nitrogen, potassium, creatinine as well as creatinine clearance as an essential part of the therapy. Recently, studies have shown that liposomal amphotericin B and fluconazole, caspofungin can be combined with each other. Hyperbaric oxygen therapy is believed to improve neutrophilic killing by higher oxygen delivery, low dose of heparin, anti inflammatory medicine can be used with good success [29–31].

Prognosis is dependent on multiple factors and early initiation of treatment is an important element. A multidisciplinary approach consisting of dental specialists, ENT surgeons, ophthalmologists and neurologist is critical in successful management of a patient with mucormycosis. Hence the general approach is to treat early, aggressively and with all modalities available.

## Conclusions

Mucormycosis is a severe, emergent and fatal infection requiring multidisciplinary management. It is a disease with various presentations, and sometimes its manifestations are atypical. For ophthalmologist, it should be kept in mind when dealing with the case of OAS, even exophthalmia, progressive periorbital or facial edema or necrosis, with or without involvement of cranial nerves in the patients accompanied with immunodeficiency disorders. The diagnosis relies on histological and mycological examination data. Early diagnosis and urgent antifungal treatment associated to surgery are of extreme importance for successful eradication of infection and for patient survival.

## Abbreviations

OAS: Orbital apex syndrome
ROCM: Rhino-orbital-cerebral mucormycosis
